# Evolution of polypyrrole electrode during electropolymerization and its effect on energy storage performance

**DOI:** 10.1038/s41598-026-47559-7

**Published:** 2026-04-07

**Authors:** Duy Pham, Ryan Gouafong, Jake Irvin, Ben McKinney, Jacob Dileonardi, Mason Cox, Ashish Aphale

**Affiliations:** 1 Department of Mechanical Engineering, Kennesaw State University, 1100 South Marietta Pkwy SE, 840 Polytechnic Ln, Marietta, GA 30060 USA; 2 Department of Mechatronics, Kennesaw State University, 1100 South Marietta Pkwy SE, Marietta, GA 30060 USA

**Keywords:** Supercapacitor, Polypyrrole, Electropolymerization, Raman spectroscopy, Electrochemistry, Chemistry, Energy science and technology, Materials science

## Abstract

This study investigates the influence of electropolymerization time on the evolution of morphology and the electrochemical performance of polypyrrole (PPy) electrodes. The PPy films were synthesized by varying the deposition time from 300 seconds to 1800 seconds, corresponding to 5 to 50 cycles. The electrodes were characterized by Raman spectroscopy, XPS, and SEM, and electrochemical analyses were conducted using cyclic voltammetry (CV), electrochemical impedance spectroscopy (EIS), and galvanostatic charge-discharge (GCD). Experimental results showed that increasing deposition time yields large cauliflower-like structures, thereby enhancing capacitance. The 50 cycle electrode exhibited the highest specific capacitance of 412 F/g in a three-electrode configuration using 1.2 M KCl aqueous electrolyte. The EIS analysis revealed a low ohmic resistance and increasing non-ohmic resistance with deposition, which may indicate the onset of diffusion-limited behavior. A symmetric PPy supercapacitor assembled using optimized electrodes delivered an energy density (ED) of 10.29 Wh/kg and power density (PD) of 146 W/kg at 0.1 A/g. These findings show that electrodeposition time plays an important role in PPy film growth, ion transport, and the resulting electrochemical performance.

## Introduction

With rising energy demand and the world’s expanding carbon footprint, there is a need to develop robust energy storage systems^1^. SC have gained significant attention in recent years due to their capability to deliver high energy and power densities simultaneously^1,2^. SCs bridge the gap between energy storage systems, such as large-capacity batteries and high-power-density dielectric capacitors^3^. When integrated into the same system for applications such as electric vehicles and power grids, high-power delivery is quickly achieved, thereby reducing the load on the battery^4^. Improvements in SC technology can lead to increased energy storage capacity, longer lifespan, and faster charging rates, which are crucial for meeting the growing energy demands^3^.

An SC, also known as an ultracapacitor, is a two-electrode device that uses a porous membrane, called a separator, and is coupled to an electrolyte solution. Common SCs include electric double-layer capacitors (EDLCs) and pseudocapacitors, which differ in the electrode materials used and the charge storage mechanisms employed, such as non-faradaic and faradaic charge transport^3,5,6^. EDLCs use carbon-based nanomaterials that enable fast non-faradic reactions, while pseudocapacitors rely on transition-metal oxides or electrically conducting polymers (ECPs) with reversible faradic reactions^7,8^. ECPs are promising materials for high-performance SCs, as they exhibit high specific capacitance and high electrical conductivity in the charged state^9^. While electrodes fabricated from metal oxides exhibit robust performance, ECPs offer advantages such as a high energy storage capacity, environmental benefits, and more affordable fabrication^10^. Amongst several conducting polymers, polypyrrole (PPy) is one of the most extensively studied polymers due to the ease of oxidation of its monomer (pyrrole), its relatively large water solubility, high conductivity, good redox (reduction/oxidation) reversibility, and environmental stability^3^.

SC performance can be enhanced by achieving a high surface area using nanofeatures that enable high porosity in electrode materials^11,12^. Several studies have shown that excellent performance from nanostructured electrodes, such as nanoparticles, nanowires, nanotubes, and nanobricks, can be achieved in various electrolyte solutions^2,11^. The morphology of the PPy electrodes largely depends on the nature of the deposition technique used. For example, the high surface area and the porous structure are typically influenced by chemical oxidative polymerization and electrochemical polymerization. Factors that affect chemical oxidative polymerization include the solvent, oxidizing agent, temperature, and time^13,14^. On the other hand, electrochemical polymerization directly deposits PPy to the desired thickness by adjusting several variables such as time, current density, potential range, scan rate, and electrolyte concentration^1,9,15,16^. Studies have shown significant differences in electrode morphology, and the resulting performance is achieved based on the above conditions. For example, Dubal et al. demonstrated that different nanofeatures, such as nanobelts, nanobricks, and nanosheets, can be formed by varying the deposition scan rate^2^. Their work shows that deposition at 50 mV/s led to the formation of nanobelt-shaped PPy, 100 mV/s to nanobrick-shaped PPy, and 200 mV/s to nanosheets. As a result, an upward trend in specific capacitance was observed, increasing from 296 F/g to 584 F/g^1^.

In this work, we examine the role of deposition time in determining the number of cycles required to understand the evolution of PPy electrodes as they deposit on the substrate. Previous studies have focused on understanding the electrochemical performance of PPy through the dopant type^17,18^, scan rate-dependent studies^2^, nanocomposite formation using carbon structures and metal oxides^20^, and the influence of different substrates on electrode performance. Moreover, several works have reported analyzing the role of thickness and material composition on the performance of PPy, for example, Wang et al^21^. demonstrated that certain dopants, such as Cl^-^, SO^2-^_4_, and 4 toluene sulfonic ions, influence high crystallinity in PPy structure, leading to poor specific capacitance with thickness due to restricted ion diffusion. Alternatively, prior work on metal-PPy nanocomposites^22^(e.g., Au/PPy^23^, Pt/PPy^24^, and Cu/PPy^25,26^) has demonstrated that increasing the thickness decreases nucleation site density and tends to increase metal agglomeration, limiting performance. Collectively, these important works show that PPy thickness strongly affects the presence of other active materials; they do not address how pure PPy itself evolves during polymerization and how its structure, ion diffusion, and electrochemical performance change as a function of deposition cycle. Despite widespread reports on PPy, there remains a limited understanding of the time-dependent growth of PPy during electropolymerization and its effect on electrochemical performance. As the polymer film grows thicker with increasing deposition cycles, it alters ion diffusion pathways, charge transport, and both ohmic and non-ohmic resistance, significantly influencing the electrochemical response. Therefore, this work investigates the time-dependent evolution of PPy films, providing insights into film growth and its influence on ion diffusion, resistance changes, and overall electrochemical performance. The electrochemical properties of PPy electrodes were tested in an aqueous 1.2 M KCl electrolyte solution using CV, EIS, and GCD. Results show that higher cycles (50 cycles) of polymer deposition yield the highest specific capacitance of 412 F/g in the three-electrode setup and 48.4 F/g from the symmetric SC button cell, with both tests conducted at 5 mV/s. Furthermore, EIS results reveal low solution resistance (R_s_) across all cycles, indicating good ionic conductivity, with an exponential increase in non-ohmic resistance (R_ct_). Diffusion behavior also significantly evolves with film thickness, starting with restricted diffusion at 5 cycles and transitioning to Warburg diffusion at higher cycles. These variations in deposition duration led to significant changes in PPy particle size and electrode performance.

## Results

### Surface morphological & chemical characterization

**Fig. 1:**
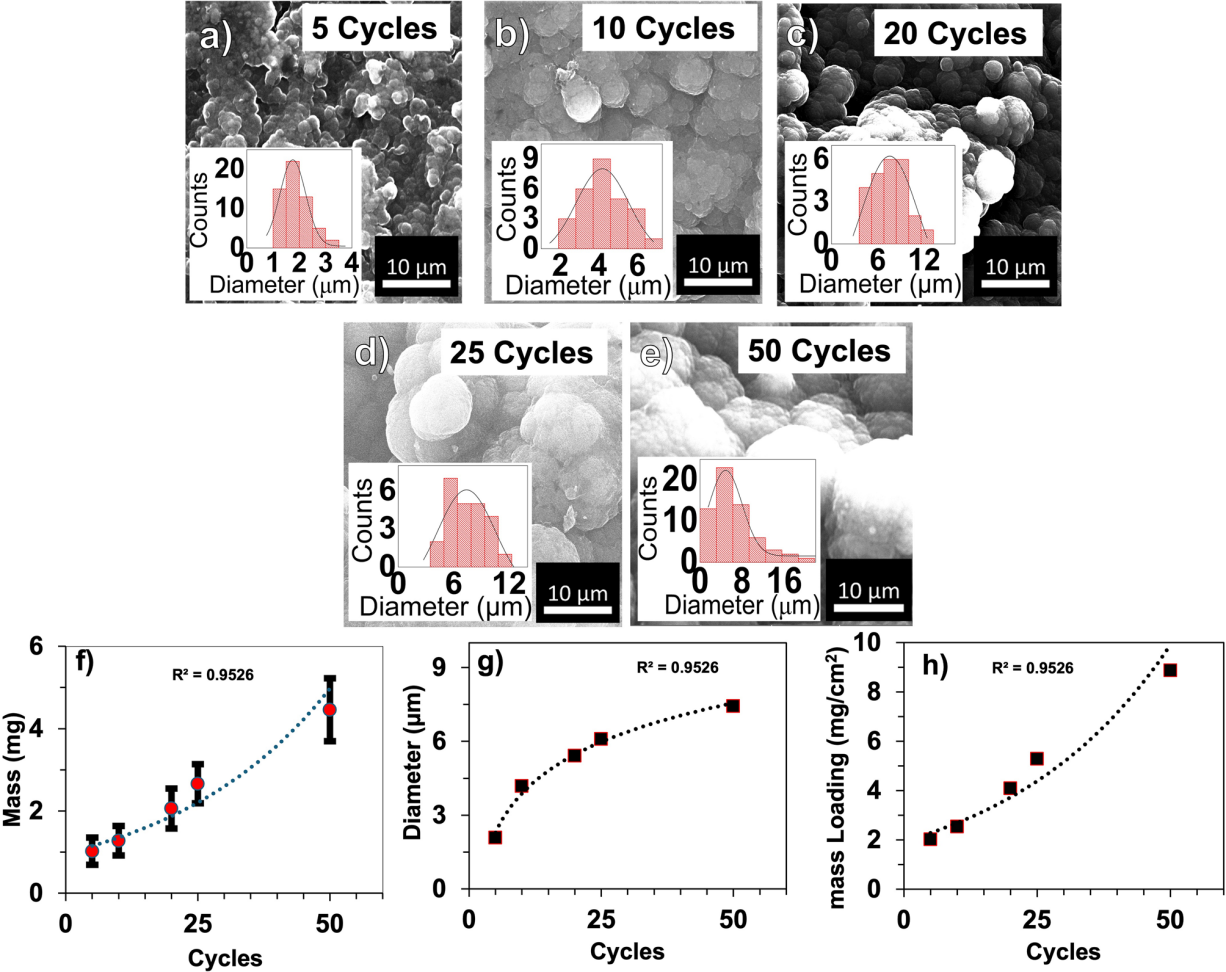
Surface morphology and physical characterization of electropolymerized PPy film as a function of deposition cycles. SEM micrograph of: (**a**) 5 cycles, (**b**) 10 cycles, (**c**) 20 cycles, (**d**) 25 cycles, (**e**) 50 cycles, (**f**) average mass of PPy, (**g**) average diameter of PPy, and (**h**) mass loading of various cycles. All SEM micrographs (a-e) were acquired at 30kV and 5000x magnification.

Figure 1 shows SEM micrographs for all PPy electrodes, uniformly covered on the surface of the graphite substrate. It is evident that the number of PPy deposition cycles on the graphite affects the structural morphology. The PPy has a relatively uneven surface that appears as circular pebbles at lower deposition cycles, which eventually agglomerate into a large cauliflower-like structure at higher deposition cycles. Lower deposition cycles, such as 5 and 10, show an overall symmetrical distribution of PPy cluster diameters. The diameter of the 5-cycle electrode is 1 to 2.35 µm. 10 cycles have a wider diameter range from 2 µm to 6 µm. Intermediate cycles of 20 and 25 demonstrate bigger diameters in the range of 3–12 µm. This is the number of cycles where the surface PPy diameter growth begins to saturate. The 50 cycle shows the largest diameter of up to 21.7 µm. These diameters indicate PPy nucleation, which may increase the surface area and porosity of the overall structure. The mass shows exponential growth: 5 cycles weigh an average of 0.8 mg, and 50 cycles weigh 4.8 mg. This suggest that after reaching approximately 3–12 µm in diameter, a second growth layer may be forming, covering the peaks of slower nucleating particles.

**Fig. 2:**
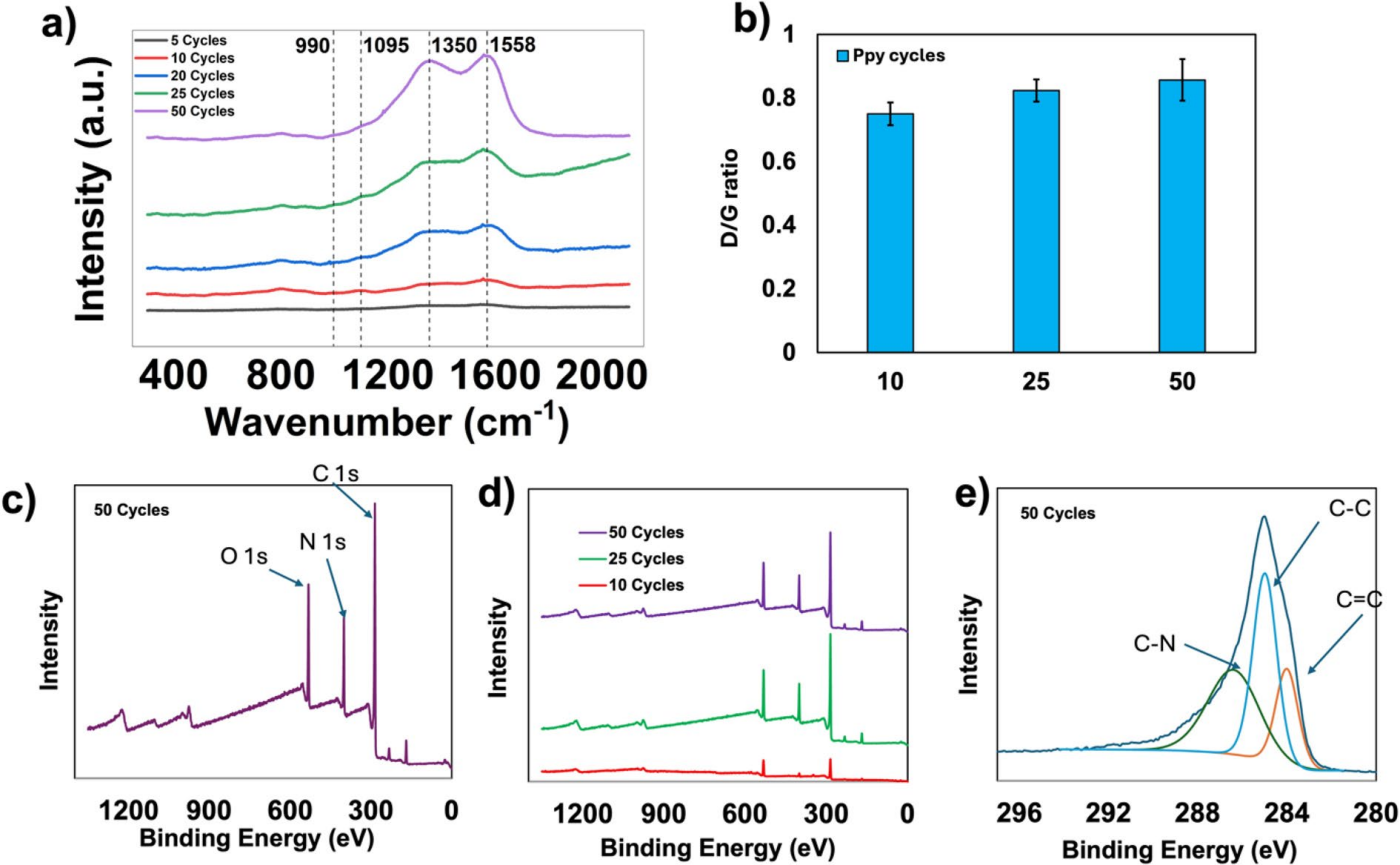
(**a**) Raman spectra of 5–50 PPy deposition cycles, (**b**) statistical representation of D/G ratio of 10, 25, and 50 cycles **c)** XPS spectra of 10, 25, and 50 cycles, (**d**) XPS spectra of 50 cycles, (**e**) deconvolution of C 1 s for 50 cycles.

The Raman spectra shown in Fig. 2a indicate similarities among the PPy evolution cycles. The peaks from the 50 cycle sample show a band at around 1558 cm^−1^, which represents the PPy C=C backbone stretching^27,28^. Bands with peaks between 1350 cm^−1^are assigned to the ring stretching mode of PPy^29,30^. Additionally, C-H in-plane deformation is characterized at bands 1095 cm^−1^ with another peak at 990 cm^−1^, depicting the pyrrole ring in-plane deformation^31,32^. Moreover, the set of peaks at 1558 cm^-1^ and 1350 cm^−1^is associated with the D-band, which corresponds to structural defects, or disorders, and the G-band (representing the vibration of highly ordered sp^2^-hybridized carbon), respectively^33,34^. The Raman D/G intensity ratio (I_D_/I_G_) has been widely accepted as a metric for quantifying structural disorder^35^. The I_D_/I_G_ of the PPy samples are 0.78, 0.73, 0.83, 0.81, and 0.92 for 5, 10, 20, 25, and 50 cycles, respectively. This shows a moderate increase in defect density. Fig 2B shows statistical data on variations in I_D_/I_G_ ratios for 10, 25, and 50 cycle samples. A shift of up to ~20 cm^−1^in the characteristic D and G bands is observed with increasing deposition time. The intensity of each peak increases with the electrodeposition time, confirming the nucleation of PPy. In essence, the observation suggests that the material transitions from having a greater number of basic C=C bonds in earlier stages to a more defined polymer structure dominated by PPy rings at later stages.

X-ray photoelectron spectroscopy (XPS) was performed to deconvolute the typical peaks observed in PPy. Here, Fig. 2c shows the overall PPy spectrum for 10, 25, and 50 cycles. There are O 1 s, N 1 s, and C 1 s signals that are typically observed in PPy spectra, with corresponding atomic percentages of 12.44%, 62.18%, and 12.53%. The N 1 s peak contains N-H, and the O 1 s has C-O bonds. The intensity count of these peaks changes drastically from lower cycles (10) to (25), where the C 1 s peak increases by a ratio of 4:1. The PPy structure is C4H5N; therefore, peaks at C 1 s were fitted to deconvolute the various carbon bonds that give PPy its unique structure. This deconvolution is shown in Fig 2d, where (C-N) is at 286.38 eV, (C-C) at 284.88 eV, and (C=C) at 283.87 eV.

### Cyclic voltammetry (CV)

**Fig. 3:**
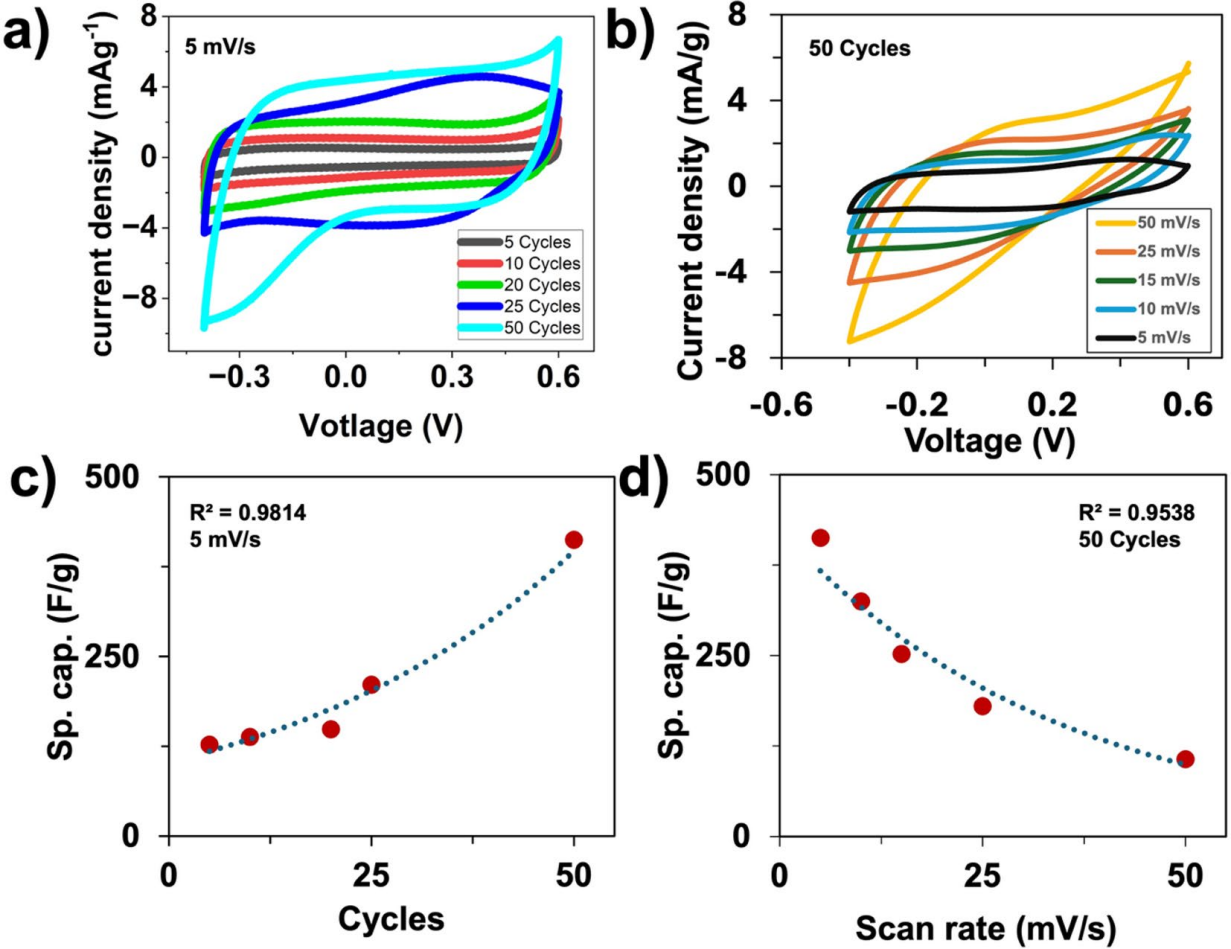
Three-electrode analysis of (**a**) CV at 5 mV/s from 5–50 cycles, (**b**) CV of 50 cycles at 5–50 mV/s, (**c**) Specific capacitance of all cycles at 5 mV/s, and (**d**) Specific capacitance of 50 cycles at 5–50 mV/s.

Electrochemical measurements were conducted to investigate the effect of electropolymerization time on PPy behavior. The electrochemical testing was conducted using a 3-electrode system in 1.2 M KCl electrolyte, yielding quasi-rectangular CV curves. Figure 3a shows that the CV curves recorded at 5 mV/s for PPy electrodes synthesized with 5 to 50 deposition cycles. The enclosed CV area becomes larger with increasing cycles, indicating higher charge storage capacity. This improvement results from the thicker PPy films formed at higher cycles, which provide a greater amount of electroactive material and more accessible redox active sites. As a result, electrodes with higher deposition cycles exhibit enhanced pseudocapacitive behavior, suggesting improved electrochemical performance. Moreover, Raman spectroscopy suggests that increasing the PPy polymerization time correlates with a higher concentration of bipolarons, which appears to contribute to the observed enhancement in capacitance. Fig. 3b shows a cyclic voltammogram of a 50 cycle PPy electrode measured from 5 to 50 mV/s scan rate. This behavior is characteristic of pseudocapacitive materials. The peak shift and increased steepness are related to the kinetically limited ion diffusion process in the PPy film. At lower scan rates (5 mV/s), ions have sufficient time to diffuse through the 50 cycle PPy film. This results in an anodic peak appearing between 0.3 and 0.5 V close to equilibrium. However, as the scan rate increases to 10 mV/s, ion diffusion becomes kinetically limited, resulting in a rightward shift of the peak and increased steepness. At higher scan rates, sluggish ion transport prevents the system from maintaining equilibrium, thereby requiring additional overpotential and an anodic shift to the right^36^. Here, calculations are done following equation (4) to determine the specific capacitance C_S1_ (F/g) of all cycles. The overall trend appears to be that higher cycles will also yield higher specific capacitance, with 50 cycles at 5 mV/s yielding the highest C_S1_ of 412.5 F/g (Fig 3d).

### Electrochemical impedance spectroscopy (EIS)

**Fig. 4:**
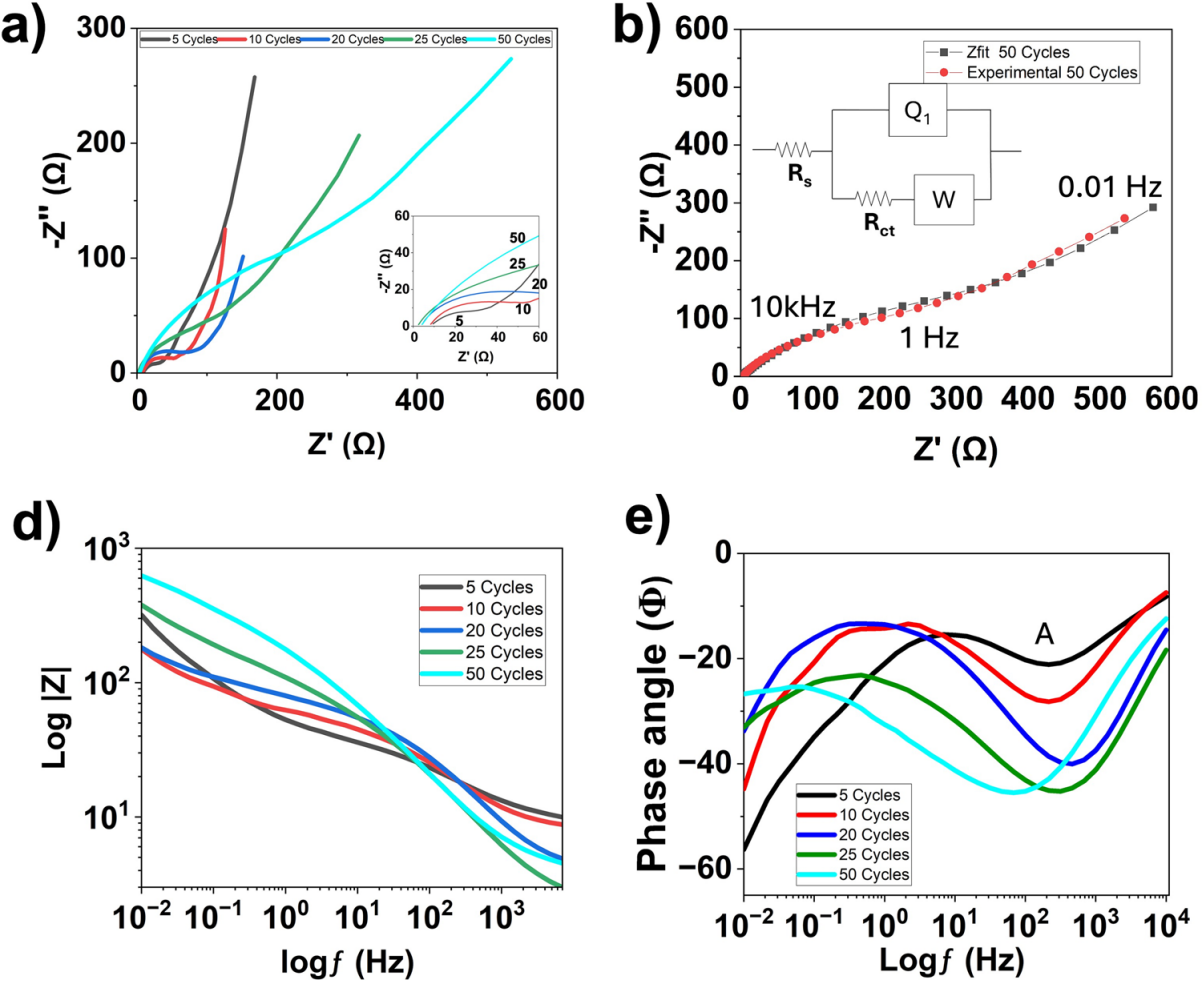
Three-electrode analysis of (**a**) EIS of all cycles, (**inset**) zoomed in EIS of all cycles, (**b**) Fitted and experimental 50 cycles with ESR, (**d**) Impedance of Bode plot, (**e**) Phase shift of Bode plot.

An EIS study was conducted in a three-electrode configuration to determine electrode impedance over a frequency range of 0.01 Hz to 10 kHz. The general equivalent series resistance (ESR) model (Fig. 4b) contains the circuit element at the electrolyte-to-surface interface known as ohmic resistance (R_s_), the semi-circle (R_ct_) in the mid frequency represents charge transfer resistance, and Q_1_ represents the constant phase element for diffusion behavior to capacitive behavior of ions on the rough surface of the PPy electrode. The intersection with the real Z’-axis in the Nyquist plot is the solution resistance (Rs), which includes the total resistance: the intrinsic resistance of the electrode materials, the electrolyte, and the contact resistance at the interfaces between the electrodes, the electrolyte, and the current collector substrates. The fitting of the Nyquist plot was performed with a fitting program ZFIT/EC-Lab version 11.52 using the equivalent circuit shown in Fig. 4b. Table 1 summarizes all the parameters obtained from the fitting. The X/√N is used to represent the error, where N is the number of data points, and X/√N is a normalized expression of X^2^, whose value is independent of the number of points and is represented as the error. The ESR model demonstrates a parallel circuit of Q_1_ and R_ct_, with the Warburg circuit element (W). The impedance of a constant-phase element is given by the equation below.

1 $$Z\left( \omega \right) = \frac{1}{{Q_{1} }}\left( {j\omega } \right)^{ - \alpha }$$

**Table 1:** EIS data table for 5–50 cycles of PPy.

Cycle #	R_s_	R_ct_	Q_1_(S^(a-1)^)	α	X/$\surd$N
5	9.28	22.93	0.000529	0.67	0.923
10	7.64	50.98	0.000702	0.61	1.152
20	1.586	83.16	0.000919	0.54	1.11
25	0.226	148.2	0.001522	0.53	0.88
50	1.191	450.6	0.001548	0.52	1.875

Where Q_1_ is admittance in S•sn, and ω is the frequency in Hz, α is a unitless parameter. As $\alpha$ approaches to 0 the CPE behaves as an ideal resistor, as α approaches close to 0.5 the CPE becomes more of a Warburg element, and as α approaches 1 the element behaves as an ideal double-layer capacitor^37^. The α values in Table 1 indicate that 5 cycle electrode (α=0.67) displayed non-ideal behavior, likely resulting from surface roughness created by immature PPy nucleation. For 10 cycle electrode (α = 0.61), the reduction in α suggests an improvement in diffusion. At higher deposited cycles from 20 to 50, as α approaches to ~0.5, indicating improvement of electrode structure and onset of Warburg-like diffusion behavior. To complement the tabulated values, the bode plot in Fig. 4d shows an overall trend of increasing total impedance with decreasing frequency. In Fig. 4e, the first angle variation in each cycle corresponds to the parallel circuit of Q_1_ and R_ct_. The next angle variation occurs in the higher-frequency range, where it shows the diffusion impedance^38^. In Fig 4e, the high-frequency peak (peak A) corresponds to diffusion behavior. As the cycles increase, the phase angle intensity gradually increases and saturates at 45°, confirming enhanced Warburg diffusion at higher cycles. Where 25 and 50 cycles exhibit diffusion due to the Bode plot peaking at 45°, and cycles below 25 exhibit a transition from restricted to Warburg diffusion, where the phase angle is below 45°. Notably, for 25 cycles, diffusion occurs at 316 Hz, whereas for 50 cycles, it occurs at 68 Hz, indicating slower charge-transfer kinetics in the thicker films.

### Galvanostatic charge-discharge (GCD)

**Fig. 5:**
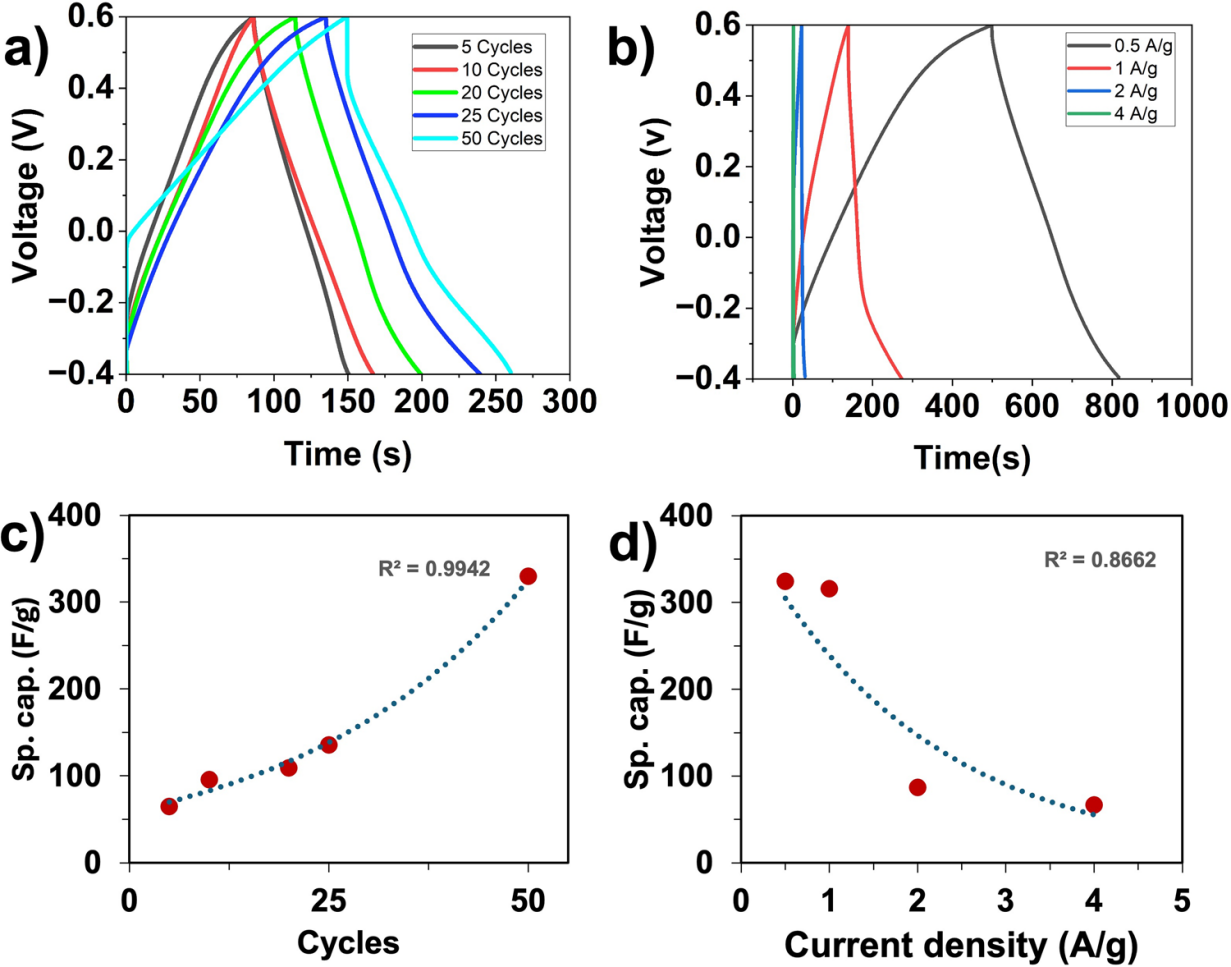
Three-electrode system of (**a**) 5–50 cycles at 1 A/g, (**b**) 50 cycles at 0.5–4 A/g, (**c**) Specific capacitance of 5–50 cycles, (**d**) Specific capacitance of 50 cycles at 0.5–4 A/g.

GCD testing was conducted at current densities ranging from 0.5 A/g to 4 A/g in 1.2 M KCl, yielding nearly symmetrical curves over 5 cycles. As more PPy is deposited, the GCD curve becomes increasingly irregular due to enhanced redox activity primarily arising from Faradaic redox processes. At lower film deposition of 5 and 10 cycle, PPy exhibits a more linear slope (triangular shape), characteristic of capacitive behavior (EDLC). However, as more PPy layers are added, the electrode introduces more complex and longer ion diffusion pathways, leading to non-uniform charge transport and enhanced redox activity. These effects collectively contribute to deviation from triangular-shaped GCD curves at higher deposition levels^39,40^. Specific capacitance was calculated for all cycles using equation (5) and is plotted in Fig. 5c. The GCD result at 1 A/g for 50 cycles yields a maximum value of 324.5 F/g. Since 50 cycles yield the best performance among the other cycles, a comparison test is shown in Fig. 5b for the 50 cycle sample, ranging from 0.5 A/g to 4 A/g. From the range of 4 A/g to 0.5 A/g, there are significantly higher charge and discharge times. Therefore, the specific capacitance is just below 100 F/g at higher current density, while at lower current density it is above 300 F/g.

### Electrochemical analysis of the SC button cell

**Fig. 6:**
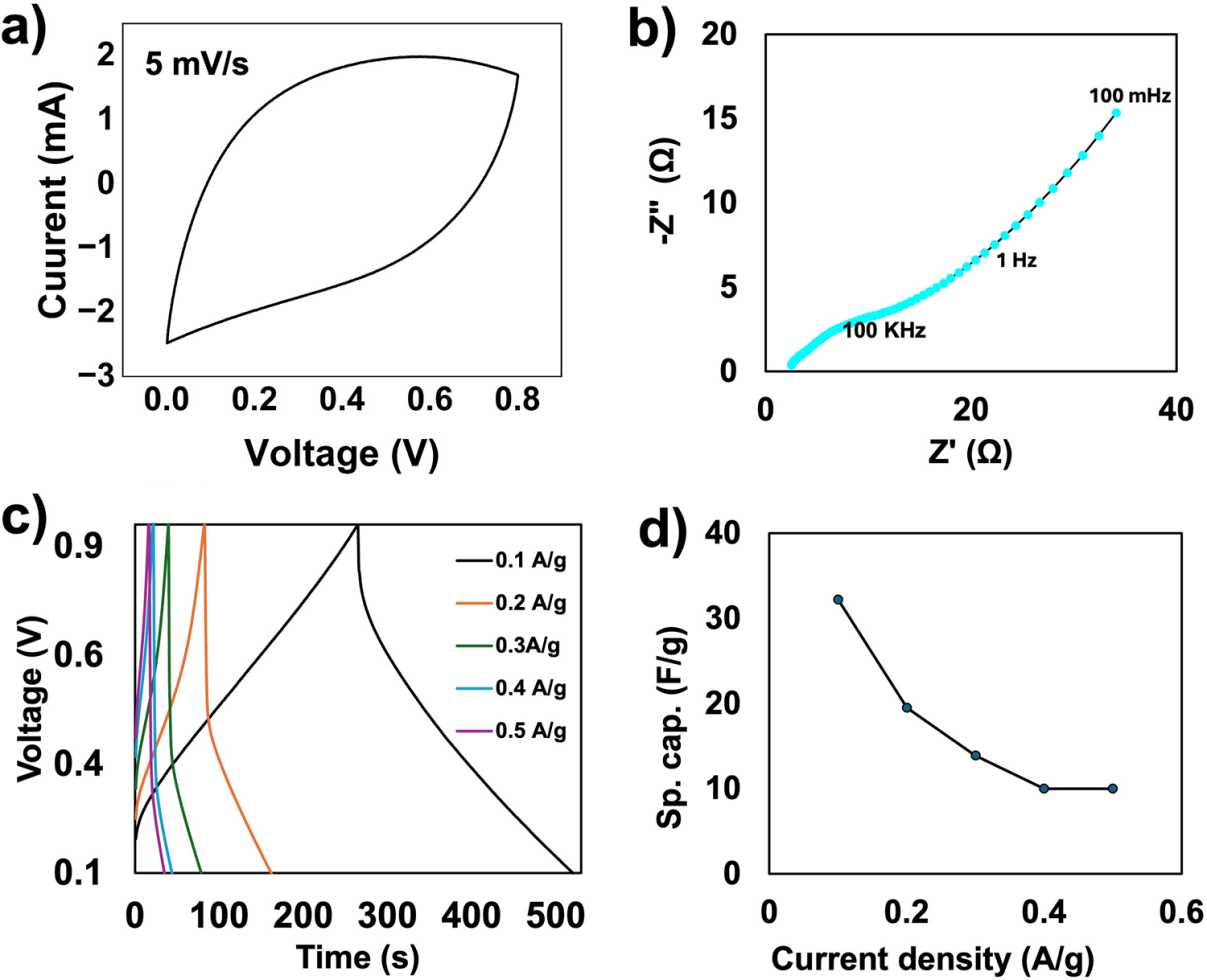
Electrochemical analysis of 50 cycles PPy button cell, (**a**) CV at 5mV/s, (**b**) Nyquist plot, (**c**) GCD curve at 0.1 A/g, (**d**) Specific capacitances calculated at different current densities.

Additionally, an SC button cell was assembled using 50 cycle sample electrodes to investigate its performance and understand how the PPy electrodes perform in an actual energy storage device. CV testing was performed at a scan rate of 5 mV/s over a potential window from 0 to 0.8 V to determine the amount of charge it could store per unit of active mass. Fig. 6a depicts the voltammogram of the cell, which shows an almost rectangular box-like shape characteristic of ideal pseudocapacitive behavior. The specific capacitance (C_s_) was evaluated using equation (4) and found to be 48.4 F/g. The observed behavior of PPy electrodes is consistent with the 3-electrode cell studies presented earlier. Furthermore, EIS analysis was conducted to measure the ohmic resistance, charge-transfer resistance, and overall impedance characteristics of the assembled SC button cell. This test was conducted over the range of 100 kHz to 100 mHz at a constant amplitude of 10 mV. The Nyquist plot in Fig. 6b shows an ohmic resistance, R_s,_ of 2.4 Ω, indicative of good ionic conductivity in the SC button cell.

Additionally, Fig. 6c shows the GCD curves conducted at current densities of 0.1, 0.2, 0.3, 0.4, and 0.5 A/g. There is a noticeable difference between the performance at 0.1 A/g and 0.2 A/g, indicating the button cell’s superior performance under charge-discharge conditions at 0.1 A/g. Thus, it was used for testing the cell’s cycling stability. The charge and discharge times (at 0.1 A/g) were 264 s and 254 s, respectively. Based on equation (5), the specific capacitance from the GCD test was calculated to be 32.2 F/g. Additionally, Fig. 6d shows a graphical comparison of the specific capacitance values at the current densities listed above, calculated using equation (5).

## Discussion

A more in-depth analysis of EIS reveals changes in PPy during evolution from lower to higher cycles, resulting in thicker films. The R_s_ is low across all cycles due to the nature of the aqueous electrolyte resistance. The R_ct_ increases exponentially with the number of deposited cycles. In Fig. 4a, the 5-cycle sample exhibits a restricted diffusion-limited process, and intermediate cycles gradually shift towards a constant-phase-element (CPE) behavior. The frequencies at these shifts are 215.5 Hz for 5 cycles, 215.3 Hz for 10 cycles, and 453.0 Hz for 20 cycles, with a range of 0.01 Hz in the mass transfer region^41^. For higher cycles at 25 and 50, the tail of the Nyquist plot exhibits Warburg behavior due to the gradual shift to 45° that is shown in Fig. 3d. Furthermore, the diffusion coefficient for the Warburg impedances is measurable at 58.124 Ω.s^1/2^ for 25 cycles and 74.583 Ω. s^1/2^ for 50 cycles.

**Fig. 7:**
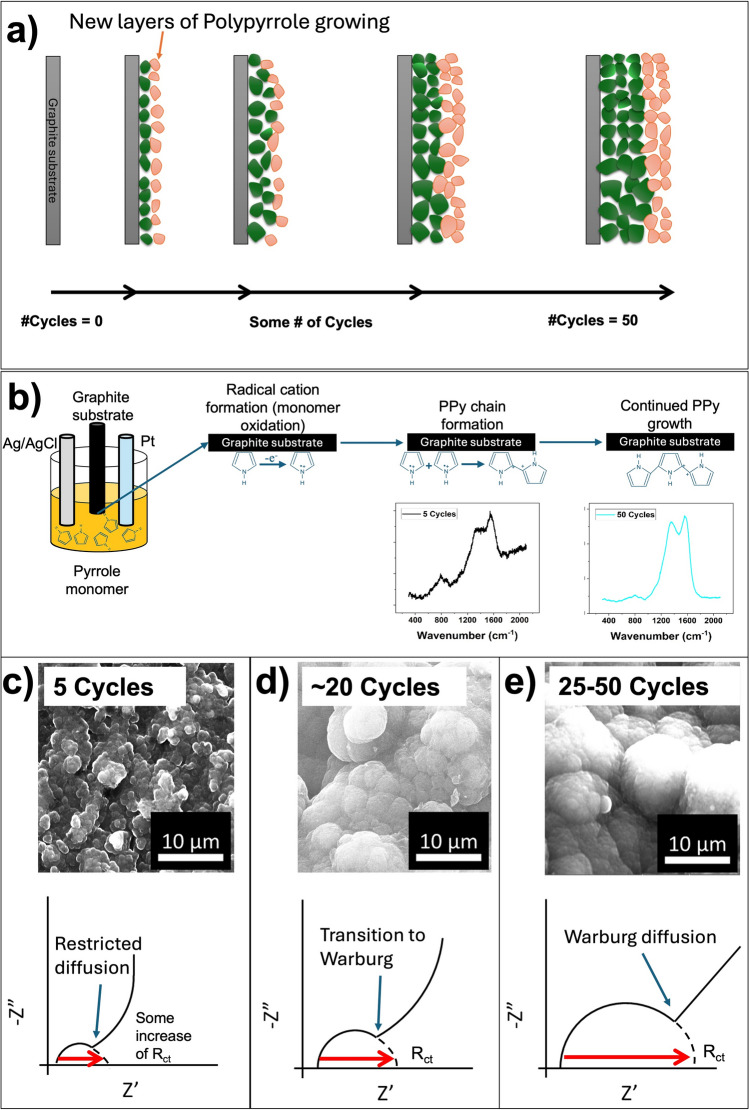
(**a**) Nucleation of PPy as a function of time (cycles), (**b**) PPy evolution, (**c**) 5 Cycles with restricted diffusion, (**d**) transition to Warburg diffusion at intermediate cycles, (**e**) Warburg diffusion at 25 −50 cycles.

As the number of electropolymerization cycles increases, as shown in the Nyquist plot in Fig. 4a and Table 1, a progressive increase in charge-transfer resistance is observed, indicating slower interfacial charge-transfer kinetics due to increased PPy thickness and transport limitations. This is supported by the shift in the low-frequency region, from near-vertical to a 45° angle (Warburg diffusion), indicating that it gets harder for ions to penetrate deep into the thicker PPy layer (Fig. 7a). Meanwhile, as evident from Fig. 7c-e, the larger cauliflower structure may provide more redox active sites, an increase in the amount of charge the film can hold, reflecting enhanced pseudocapacitive charge storage arising from the larger amount of electroactive PPy. It has been shown using Raman spectroscopy that as the PPy polymerization time increases, the film contains a higher concentration of bipolarons, leading to improved capacitance^29,42^This is because longer electrodeposition times lead to greater oxidation, increasing the formation of bipolarons (localized defects that create lattice distortions within the polymer structure), thereby facilitating charge-carrier mobility^43^. Similar trade-off behavior was observed by Budi et al^44^. where a composite material from polyaniline and PPy exhibited the highest performance compared to the individual composite components, despite having an R_ct_value significantly higher than polyaniline. Besharat et al^45^. showed that, with all other parameters held constant, increasing the deposition time during electropolymerization of a PPy/GO film resulted in thicker films. Chen et al^29^. demonstrated that an increase in the thickness of deposited PPy films directly correlates with an increase in defects, as measured by the Raman I_D_/I_G_ ratio. Both studies validate the observed behavior of the Raman spectra of films at each cycle group. This is also evident in Fig. 2, which shows an increase in the D-band intensity relative to the G-band as the deposition time increases. It has been posited that this defect proliferation could be attributed to the fact that some sp^2^-hybridized carbon atoms (G-band) become distorted by internal oxidation and are thus converted to sp^3^-hybridized carbon atoms (D-band) during synthesis^46^. Thus, an increase in cycles (deposition time) leads to greater oxidation, thereby increasing the number of defects. Furthermore, increased defects in the sample would lead to a corresponding increase in charge transfer resistance (R_ct_), as evidenced by the EIS analysis. In the same light, greater disorder in the PPy nanostructure creates more redox-active sites with easier ion access while maintaining reasonable conductivity, resulting in higher specific capacitance (C_s1_) as shown in the CV study with 50 cycles, which has the highest C_s1_ value. Slight shifts towards lower wavenumbers (as deposition time decreases) observed for the major peaks described in the Raman results section could indicate a switch from an oxidative (higher cycles) to a neutral (lower cycles) PPy state^27^. The significant increase in the intensity of the D and G-bands with increasing deposition time suggests that more material is deposited, creating more concentrated (ordered and stronger) bonds as the film gets thicker, which results in a stronger Raman signal observed as increased intensity^47^.

Therefore, while the R_ct_ increases with the deposition cycle, the 50 cycle PPy sample shows the highest specific capacitance among the samples studied. This is mainly due to a trade-off between redox-active material and ion-diffusion limitations, as observed in experimental results. As the PPy film grows, a larger fraction of electroactive sites becomes available, enhancing the charge storage capacity. The CV and GCD results show a higher redox peak and longer discharge times at 50 cycles, indicating that a larger portion of the film remains electrochemically accessible. The rise in R_ct_ reflects longer ion diffusion pathways in thicker films. However, a greater number of redox-active sites outweighs the diffusion limitations, leading to a net increase in capacitance.

**Fig. 8:**
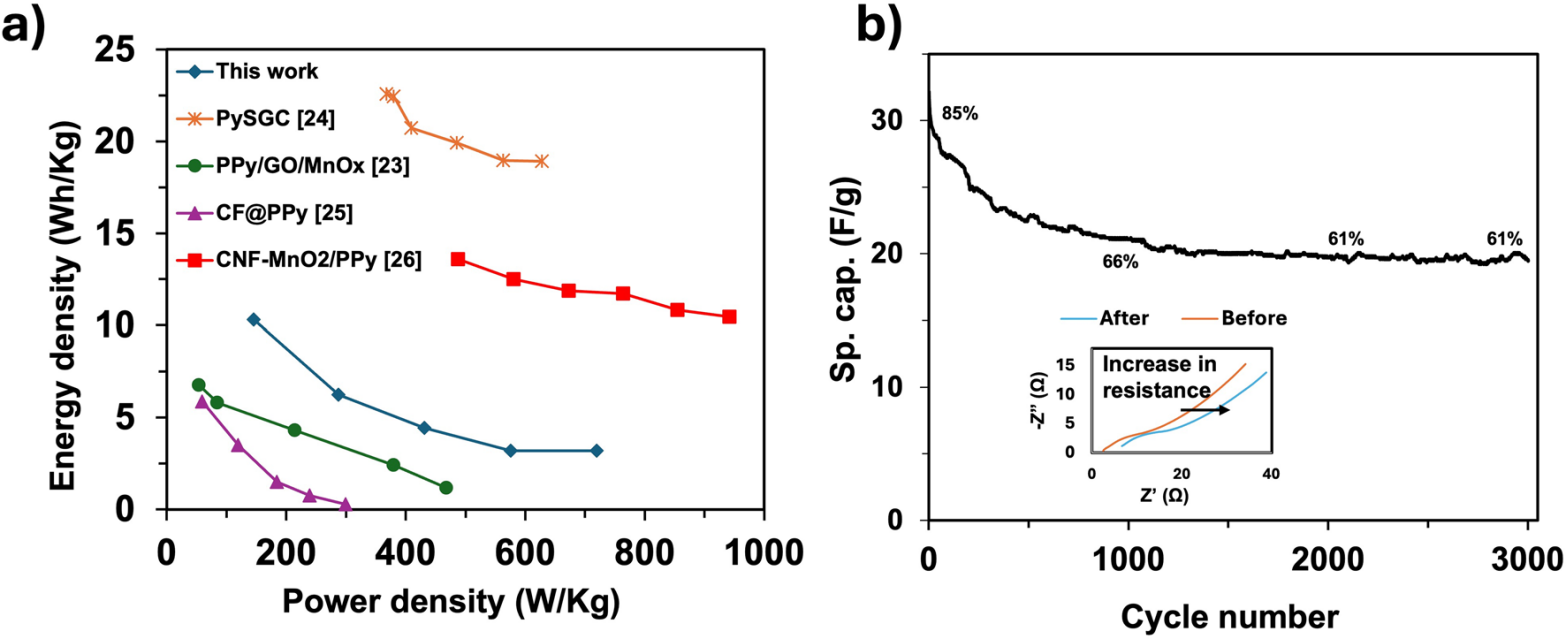
(**a**) Ragone plot of the SC button cell compared to similar studies, (**b**) Cycling data of the button cell over 3000 charge/discharge cycles with 61% capacitance retention, and postmortem EIS analysis of the button cell.

The Ragone plot is used to compare this work with other two-electrode devices, as shown in Fig. 8a. In this graph, the Ragone plot demonstrates that other SCs are made from nanocomposites that are pseudocapacitive in nature. PD and ED values of this work were derived from the SC button cell analyzed at current densities of 0.1, 0.2, 0.3, 0.4, and 0.5 A/g. The energy density (ED) and power density (PD) were calculated based on the following equations:

2 $$E = \frac{1}{2}C_{S2} \left( V \right)^{2}$$

3 $$P = \frac{E}{t}$$

Where E is energy density, P is power density, C_S2_ is gravimetric capacitance calculated from equation (5), V is the potential window during discharge, and t is the discharge time in seconds. From Fig. 8a, the corresponding ED and PD values for the button cell device are 10.29 Wh/kg and 146 W/kg, respectively, at a current density of 0.1 A/g, while at 0.2 A/g the device develops 6.23 Wh/kg and 288 W/kg. A similar trend is observed over higher current densities. This is because at lower current density values, charge-discharge times are longer, resulting in better ED. On the other hand, at higher current densities, charge-discharge times are shorter, resulting in better PD values but a drop in ED due to increased internal losses^19^. At the highest current density of 0.5 A/g, the button cell delivers 3.20 Wh/kg and 720 W/kg, supporting the previous statements. Table 2. compares this work with other studies that used PPy nanocomposites as device electrodes.

**Table 2:** Comparison against other PPy-based two-electrode button cell studies.

Device Material	Potential Window (V)	Test parameter	Electrolyte	Energy Density (Wh/kg)	Power Density (W/kg)	Reference
PPy	0.0 - 0.8	0.1 A/g	1.2 M KCl	10.29	146	This work
PySGC	0.0 - 0.8	1 A/g	1 M H_2_SO_4_	22.5	369.17	Ref^48^
PPy/GO/MO_x_	−0.2 - 0.7	2 mV/s	1 M Na_2_SO_4_	6.57	54.04	Ref^19^
CF@PPy	0.0 - 1.2	0.1 A/g	0.3 M NaClO_4_	5.87	60	Ref^49^
CNF-MnO_2_/PPy	0.0 - 1.0	0.5 A/g	1 M KCl	13.68	485.22	Ref^20^
NiCO_2_O_4_@PPy|NWAs	0.0 - 1.6	10 mV/s	3 M KOH	58.8	365	Ref^50^
DMF PS@PPy20	0.0 - 1.0	0.5 A/g	1 M H_2_SO_4_	40	490	Ref^51^
AOT/PVA-H_2_SO_4_	0.0–0.8	0.5 A/g	Gel 1 M H_2_SO_4_	14.6	174.6	Ref^52^
PPy:Ce	0.0–1.0	0.5 A/g	1 M H_2_SO_4_	21.3	8100	Ref^18^

Lim et al^19^. fabricated a SC device using synthesized PPy film, PPy/GO (graphene oxide), and PPy/GO/MnOx (manganese oxide) composites. In this study, the PPy/GO/MnOx nanocomposite-based device showed the best performance in the Ragone plot, followed by PPy/GO and then the PPy film. The samples were tested in 1 M Na2SO4 over a 0.9 V range. The variations of ED and PD densities were consistent, with the characteristic inverse relation between them. PPy/GO/MnOx and PPy/GO delivered nearly identical performance at 100 mV/s, with 1.17 Wh/kg and 468.68 W/kg, respectively. Meanwhile, at a scan rate of 2 mV/s, the inversely proportional values were 6.75 Wh/kg and 54.04 W/kg. In an attempt to analyze the effect of the anionic surfactant sodium lauryl sulfate (SLS) on enhancing the performance of PPy/rGO/Co_3_O_4_(PySGC), Athira et al^48^. synthesized the electrodes using a one-pot hydrothermal method before adding SLS. A symmetric SC device (labeled PySGC SSC) was fabricated for testing in 1 M H_2_SO_4_ over a 0.8 V potential window. The SSC displayed the highest ED of 22.5 Wh/kg with a corresponding PD of 369.17 W/kg at 1 A/g, compared to the PyGC (electrode material without SLS) device, which delivered EC and PD of 20.8 Wh/kg and 340.4 W/kg, respectively. On the other hand, the SSC PySGC maintained an ED of 18.9 Wh/kg and PD of 632.46 W/kg at 6 A/g.

Another study tested a flexible solid-state (FSC) fabricated from a carbon fiber/Polypyrrole (CF@PPy) electrode, synthesized by electropolymerization, with a PVA/H_3_PO_4_ gel electrolyte^49^. The electrochemical analysis of the FSC was performed over a high potential window of 1.2 V. A maximum ED of 5.87 Wh/kg was obtained at PD of 60.0 W/kg from a current density of 0.1 A/g, whereas, at 0.5 A/g, the FSC delivered a maximum PD of 299.93 W/kg, maintaining an ED of 0.27 Wh/kg.

Moreover, Mohd et al^20^. performed a two-electrode electrochemical analysis on PPy coated on manganese oxide-carbon fiber (CNF-MnO_2_/PPy) synthesized using electrospinning, carburization, and in-situ polymerization. This analysis was performed in 1M KCl over a potential window of 1.0 V. The lowest current density of 0.5 A/g delivered an ED of 13.68 Wh/kg with an equivalent PD of 485.22 W/kg. At the highest current density of 1.0 A/g, the ED and PD were: 10.4 Wh/kg and 942.52 W/kg, respectively. Although with varied ED and PD values, each of these studies shows a steady relationship between PD and ED across the test parameter ranges, and the characteristic trade-off between ED and PD, as shown in Fig. 8.

Other studies report high values, following a similar trend, the composite material nickel cobaltite-PPy nanowire arrays (58.8 Wh/kg and 365 W/kg), polystyrene@PPy prepared with N, N-dimethylformamide (DMF) etch solvent with shell thickness of 20 nm (40 Wh/kg and 490 W/kg), interfacial polymerized PPy (14.6 Wh/kg and 174.6 W/kg), and cerium-doped PPy nanofibers (21.3 Wh/kg and 8.1 kW/kg).

The long-term stability of the SC button cell was examined using GCD measurements over 3000 charge/discharge cycles at 0.1 A/g. Fig. 8b illustrates the variation in specific capacitance of the SC button cell and retention percentage over 3000 cycles. The cell retained about 85% capacitance for 100 cycles and 61% after 3000 cycles. The button cell showed moderate capacitance retention, despite the inherently poor mechanical cycling stability of PPy, which may be attributed to degradation of the polymer chains and active electrode materials due to excessive swelling and shrinking of the polymer during charge/discharge cycles^53,54^. The postmortem electrochemical analysis of the button cell was performed using CV and EIS, as shown in Fig. 8b. The measured specific capacitance was 32.7 F/g after 3000 cycles. Moreover, the postmortem EIS analysis shows an increase in the device’s ohmic resistance from 2.4 Ω to 6.7 Ω. These observations suggest deterioration in the charge-storing capacity and in interactions at the electrode/electrolyte interface of the button cell. According to^55,56^, increases of 20% or more in capacitance and 100% or more in ohmic resistance are associated with deterioration in button cell performance. The main factors responsible for these observations include poor adhesion between the electrode and current collector due to swelling and shrinking, thereby increasing contact resistance^57,58^, changes in porosity of the electrodes due to pores being blocked by absorbed and/or deposited ion species^56^, and reduction in the electronic conductivity of PPy as the number of cycles increases^59^.

## Methods

### Materials

Sodium Sulfate (reagent, 99%), Pyrrole (reagent, 98%), and distilled water were purchased from Sigma-Aldrich. LIR2032 button cell assembly set was purchased from MTI Corp. All materials used within the experiment and polymerization process were used as received. The solution, comprising 3.1% (v/v) aqueous pyrrole, 1.5% (w/v) sodium sulfate, and distilled water, was mixed thoroughly using a magnetic stirrer for at least 15 min at room temperature. Pyrrole was electrochemically deposited on a graphite substrate. The substrate was cleaned with DI water and isopropyl alcohol (IPA) before use to avoid any contamination. A standard procedure was developed to polish the substrate surface using 800-grit polishing paper at 400 rpm to achieve uniform surface conditions before deposition.

**Fig. 9:**
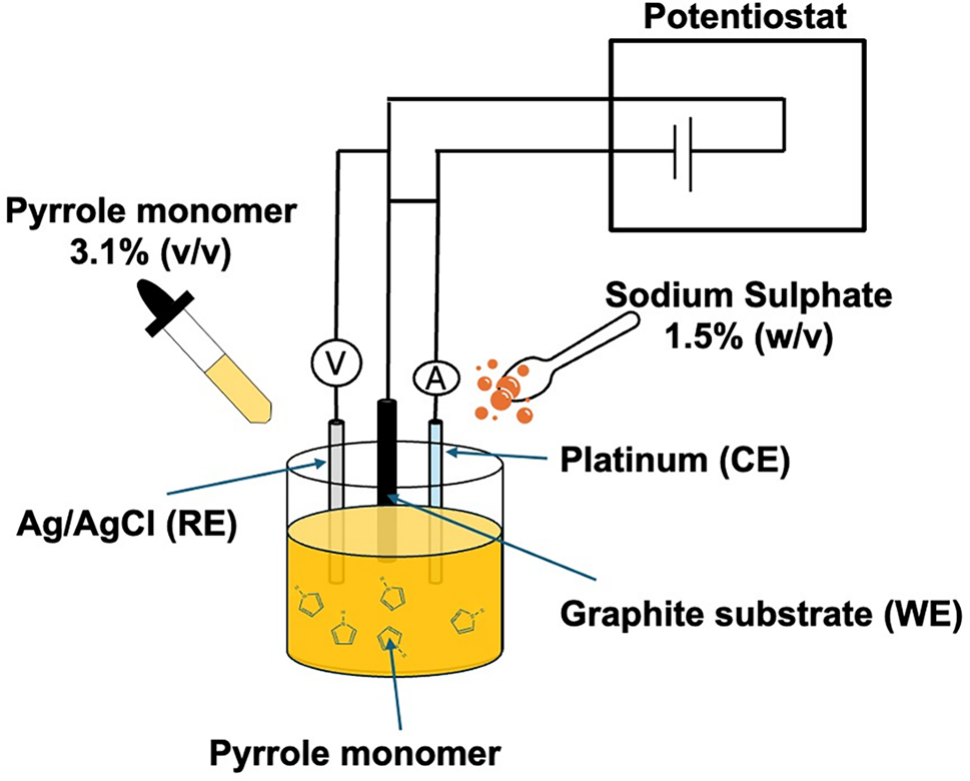
Schematic illustration of electrochemical polymerization of Pyrrole monomer on graphite substrate.

### Electrode synthesis

A three-electrode setup was used for the electropolymerization of Pyrrole. The graphite substrate was used as the working electrode (WE), platinum (Pt) as the counter electrode (CE), and a leakless silver/silver chloride (Ag/AgCl) reference electrode (RE) (eDaq Inc.). The working electrode for this experiment was a graphite rod 8 mm in diameter. The active area for electropolymerization was located on the top flat surface of the graphite substrate with a circular surface area of 50.3 mm^2^. Electropolymerization was conducted over a potential range of 800 mV to 900 mV at a scan rate of 5 mV/s. Several electrodes were synthesized by varying the number of deposition cycles: 5, 10, 20, 25, and 50, with mass loadings of 2.02, 2.52, 4.10, 5.29, and 8.73 mg.cm^-1^, respectively. Additionally, using the same setup and parameters, a PPy film (50 cycles) was synthesized on a 0.13mm thick graphite foil and used as electrodes for the two-electrode SC button cell, Fig. 9.

**Fig. 10:**
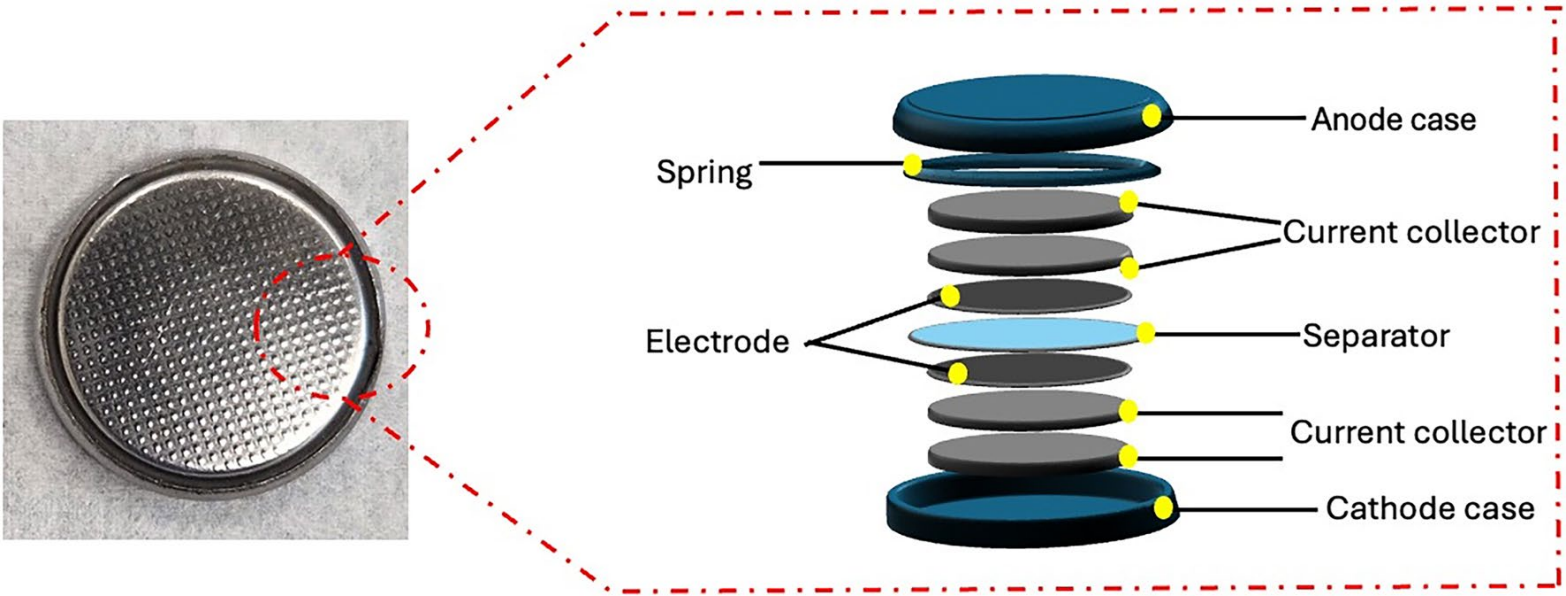
Exploded-view illustration of the fabricated button cell, showing the arrangement and structure of all device components.

After deposition, electrodes were rinsed with DI water and dried at room temperature overnight for further characterization. The mass of the deposited electrode material was determined by weighing the electrodes (US Solid analytical balance, 0.1 mg) before and after deposition onto the graphite substrate, after they were dried overnight.

### Morphological and chemical characterization

The surface morphology of the electrodes was characterized using a scanning electron microscope (SEM) (Tescan Vega 3 at 30 kV). Raman spectroscopy was performed on a Renishaw spectrometer with a 532nm green laser excitation source. Additional experimental parameters included 100% laser power and a 30-second testing time over 6 accumulations. X-ray photoelectron spectroscopy (XPS) is performed using a Thermo K-Alpha instrument with an Al Kα source and a spot size of 400 µm.

### Electrochemical characterization

The electrochemical performance of PPy was tested using both three-electrode configurations in 1.2 M KCL. As the primary objective of this study was to evaluate the electrochemical performance of the PPy electrode material rather than optimize the electrolyte composition, 1.2 M KCl electrolyte is used for consistency, as it provides a neutral electrochemical environment that minimizes electrolyte-based changes in the electrode and avoids corrosion of the button cell, as often observed in other electrolytes such as H_2_SO_4_^1^. The samples were analyzed using the Potentiostat Zive SP1 with a CV potential window from −0.4 V to 0.6 V at various scan rates of 5, 10, 20, 25, and 50 mV/s. EIS was conducted in the range of 10 kHz to 0.1 mHz for a three-electrode system with an amplitude of 5 mV a.c, and galvanostatic charge-discharge (GCD) was recorded as well at current densities ranging from 0.5 to 4 A/g. For the two and three-electrode setups, the specific capacitance values were calculated from the cyclic voltammogram (CV) graphs using the following equation^60^:

4 $$C_{S1} = \frac{1}{vm\Delta V}\smallint IdV$$

To determine the total capacitance, the area under the CV curve was integrated. Where C_S1_ is the specific capacitance (F/g), m is the mass of the active electrode material (g), ∆V is the potential difference, and *v* is the scan rate (mV/s).

From GCD, the specific capacitance (F/g) values were calculated from the following equation:

5 $$C_{S2} = \frac{I\Delta t}{{m\Delta V}}$$

Where I is the current in amps (A), ∆t is the time (s) after charging, ∆V is the potential window in voltage (V) after IR drop, and m is the mass (g). The total mass of both electrodes in the SC button cell was considered when calculating the capacitance using equations (4) and (5).

### SC button cell assembly

To assemble the SC button cell, the electrodes were polymerized onto a 0.13 mm-thick, 15.5 mm-diameter graphite foil. The active mass of the electrodes was measured to be 11.1 mg. LIR2032 anode and cathode casings were used as outer cell parts. Both sides had a component arrangement of SS316 (0.2mm thick) and SS304 (0.5mm thick), with the PPy electrode separated by a filter paper soaked in 1.2 M KCl electrolyte. A 1.1 mm spring was placed against the anode case to keep tension in the button cell. The assembly was crimped to get the final button cell device, Fig. 10.

## Conclusions

This work demonstrates that electropolymerization time influences the PPy structure and the resulting electrochemical properties of electrodes. Increasing the deposition cycles produces larger films with greater mass, leading to a larger number of redox-active sites and thereby improving the charge storage capacity, as evident from CV and GCD results. Increasing the deposition cycle increases the charge-transfer resistance; the added redox-active sites compensate for this increase, leading to an overall improvement in specific capacitance. EIS analyses confirm a transition from restricted diffusion to a Warburg diffusion process as more PPy film deposits, indicating a more complex ion-transport process. A symmetric button cell configuration assembled using 50 cycle PPy electrodes shows moderate performance over 3000 cycles, retaining about 60% of its initial specific capacitance. Overall, the deposition time plays a critical role in optimizing PPy-based supercapacitor electrodes.

## Data Availability

Data is available upon request to the corresponding author.
